# Real-world effectiveness of Motivational Enhancement for Engagement in Treatment (MEET) to improve substance use disorder care transitions

**DOI:** 10.1016/j.dadr.2025.100332

**Published:** 2025-04-11

**Authors:** Brent R. Crandal, William H. Eger, Naomi Hillery, Amy Panczakiewicz, Zhun Xu, Freddy Arriola, Kelsey S. Dickson

**Affiliations:** a Department of Psychiatry, University of California San Diego, La Jolla, CA, USA; b Chadwick Center for Children & Families at Rady Children’s Hospital, San Diego, CA, USA; c School of Social Work, San Diego State University, San Diego, CA, USA; d School of Medicine, University of California San Diego, La Jolla, CA, USA; e Health Services Research Center, Herbert Wertheim School of Public Health, University of California San Diego, La Jolla, CA, USA; f Department of Child and Family Development, San Diego State University, San Diego, CA, USA; g Child and Adolescent Services Research Center, San Diego, CA, USA

**Keywords:** Substance use disorder, Continuity of care, Treatment effectiveness, Engagement-focused intervention, Behavioral health services, Pragmatic effectiveness

## Abstract

**Introduction:**

Substance use disorder (SUD) treatment effectiveness relies on service continuity during care transitions (e.g., residential to outpatient). Motivational Enhancement for Engagement in Treatment (MEET) is a structured engagement-focused intervention designed to improve service utilization. This study tests the real-world effectiveness of MEET for individuals transitioning between SUD treatment settings.

**Methods:**

Individuals receiving withdrawal management and residential SUD treatment in the San Diego County Behavioral Health Services Drug Medi-Cal Organized Delivery System between March 2021–January 2022 were included in this study. We used logistic regression via generalized estimating equations to calculate adjusted odds ratios (AORs) and 95 % confidence intervals (CIs) that accounted for clustering within treatment facilities and individuals, and stabilized inverse probability of treatment weighting (IPTW) of baseline covariates to assess the probability of connecting to step-down SUD treatment given intervention status (MEET or treatment as usual). We also assessed the probability of timely connection to step-down treatment (i.e., within 10 days of discharge).

**Results:**

Of 10,011 participants in this quasi-experimental study, 141 (1.4 %) received MEET. Participants who received MEET were more likely to be connected to SUD treatment (AOR=1.79, 95 % CI: 1.11, 2.90) and of being connected in ≤ 10 days (AOR=1.65, 95 % CI: 1.01, 2.70) than participants who did not receive the intervention.

**Conclusions:**

Among individuals with a SUD, MEET demonstrated real-world effectiveness for improving connections to step-down care, with participants receiving the intervention having nearly twice the odds of timely connection. This indicates practical engagement-based interventions can improve SUD treatment continuity across care transitions.

## Highlights

• The MEET intervention enhanced client engagement in the transition between SUD care settings.
• Individuals participating in MEET were 79 % more likely to connect to step-down SUD treatment.
• Individuals participating in MEET were 65 % more likely to connect to step-down SUD care in ≤ 10 days.
• The MEET intervention was effective when implemented practically, in real-world settings.

## 1 Introduction

Globally, substance use disorders (SUDs) are highly prevalent but relatively scarcely treated (Connery et al., 2020), persisting chronically for a substantial proportion of the population (Fleury et al., 2016). The most recent National Survey on Drug Use and Health showed three-fourths of individuals in the United States (U.S.) who might have benefited from SUD treatment did not receive it and a large portion of them did not perceive a need for it (Substance Abuse and Mental Health Services Administration, 2024). Among those who seek treatment, many leave before treatment completion (Lappan et al., 2020). This may be due to SUD treatments occurring across diverse locations (e.g., hospitals, outpatient medical care facilities, residential treatment settings, community-based support), often necessitating transitions from more intensive to less intensive care settings (Proctor et al., 2017, Krawczyk et al., 2023, Martin and Krawczyk, 2024) that often hinder continued treatment engagement that impact outcomes, with better outcomes for those who utilize long-term, continuous treatment models (Beaulieu et al., 2021, Luchansky et al., 2000, Harris et al., 2015).

Interventions have been developed to promote treatment retention in the transition between different SUD settings (Krawczyk et al., 2023), such as peer recovery coaching, patient navigation (James et al., 2023, Treitler et al., 2024) and setting treatment goals has predicted SUD treatment retention and completion in outpatient settings (Baird et al., 2023). However, evidence-based interventions to support these step-down transitions in real-world settings are early in their development (Incze et al., 2024). Motivational Interviewing (MI; Miller and Rollnick, 2012) may offer strategies to contribute to effective, practical, and replicable interventions needed to enhance the uptake and continued utilization of SUD treatment services. MI interventions leverage personal motivation for clients, particularly during transitions between levels of care (DiClemente et al., 2004, Groshkova, 2010, McKay, 2009) and can be implemented to enhance service continuity between programs and providers (Sharp et al., 2021, Naeger et al., 2016). Research on MI has demonstrated broad effectiveness and adaptability for enhancing intrinsic motivation for change, which is thought to be an important indicator of successful care utilization, including the transition between levels of care (Burke et al., 2003). There are examples of MI contributing to general retention in outpatient SUD treatment and integrating MI techniques may enhance continuity of care transitions in SUD treatment (Carroll et al., 2006). Here, we describe a community-partnered effort to implement and test the impact of an MI-based intervention, Motivational Enhancement for Engagement in Treatment (MEET), within publicly funded SUD treatment programs in San Diego County.

### 1.1 Motivational Enhancement for Engagement in Treatment (MEET) intervention

MEET is a seven-step manualized intervention designed to encourage continued utilization of treatment and services by integrating discrete MI techniques (Miller and Rollnick, 2012) with realistic planning to overcome barriers to change. MEET was initially developed and implemented as a brief telephone intervention for caregivers initiating outpatient behavioral health services within a single treatment program. Since the initial pilot, MEET has been adapted for a range of services (i.e., HIV prevention and outreach services; children, youth and family outpatient behavioral health services; SUD recovery and transition planning) and broader implementation contexts (e.g., individual providers, programs, and county-level behavioral health and SUD treatment systems) but has not yet been evaluated for effectiveness in published literature. As part of a community-initiated endeavor to improve SUD service outcomes, the MEET intervention was integrated into SUD discharge planning practices in publicly funded programs in San Diego County with the goal of improving successful and timely step-down connections to care.

The MEET intervention includes seven steps which were focused on discharge and transition planning. Intervention steps (Table 1) are designed to specify and sequence critical steps of MI and treatment planning but also what is often referred to as the “spirit of MI,” in which the client has the capacity for success and is the expert of their recovery process. Service providers follow these steps with MI communication skills such as OARS (Open-ended questions, Affirmations, Reflections, and Summaries), an ask-tell-ask approach, and a nonjudgmental attitude (Miller and Rollnick, 2012). Following these steps encourages practitioners to question, listen and explore with the client rather than teach, guide and instruct, even if the practitioner has not had extensive training in MI.

**Table 1:** MEET Intervention Steps, Actions & Purpose.

Step	Actions	Purpose
1. Introduce & Orient	Make introductions and describe the purpose for the meeting.	Develop alliance early on by prioritizing clarity for the individual, so they understand the process and purpose for the meeting and are warmly familiarized with the service provider.
2. Hear Their Story	Invite individuals to share their story, why they are seeking support, what kind of support they would like, the possible costs and benefits for change and genuinely affirming steps they have taken so far or would like to take.	Demonstrate a genuine and nonjudgmental desire to understand and respect the individual’s experiences and autonomy, with openness, accurate empathy and a willingness to roll with resistance.
3. Explore Goals	Elicit the individual’s goals, values, and reasons for change using open-ended questions. Reflect values and reasons for change. Acknowledge honesty and concerns.	Give individuals an opportunity to consider their desires for change, to appraise their future steps or growth, and uncover possible sources of hopefulness.
4. Provide Feedback: Services, Goals & Roles	With permission, provide feedback about how treatment is meant to support change, the roles involved in treatment, and how treatment steps contribute to treatment success.	Invite the individual’s consideration of how treatment might align with their goals.
5. Explore Responses to Feedback & Areas of Alignment	Explore individual’s reactions to feedback in terms of their own goals and values. Identify areas of alignment between the individual’s goals and the treatment process.	Elicit an authentic discussion about if and how treatment meets the individual’s needs right now and reinforce hopefulness or change talk.
6. Describe Process & Problem Solve Around Barriers	Identify next steps while openly discussing obstacles. Help the individual identify their own solutions.	Support the individual in realistically planning for the future and identify how the treatment can be part of that plan.
7. Provide Summary, Affirm & Next Steps	Highlight what has been said about expectations, motivation for change and treatment. Affirm, summarize, and plan for next steps.	Reflect and reinforce where the individual would like to go from here and the practitioner’s willingness to support those steps with warmth and without judgement.

**Table 2:** Characteristics of participants admitted to a treatment program within the San Diego County DMC-ODS, July 2020–January 2022.

	**Did not receive MEET (n = 9870; 98.59)**^a^	**Received MEET (n = 141; 1.41)**	**Overall (N = 10,011; 100.00)**	**P-value**^b^
**Individual-level characteristics**				
**Mean age in years (SD)**	36.79 (11.40)	36.87 (10.87)	36.79 (11.39)	0.933
**Race/Ethnicity**				0.043
White	4964 (50.29)	87 (61.70)	5051 (50.45)	
Hispanic	2981 (30.20)	38 (26.95)	3019 (30.16)	
Black/African American	1042 (10.56)	7 (4.96)	1049 (10.48)	
Other	879 (8.91)	9 (6.38)	888 (8.87)	
Missing	4 (0.04)	0 (0.00)	4 (0.04)	
**Gender**				0.239
Cisgender man	6279 (63.62)	98 (69.50)	6377 (63.70)	
Cisgender woman	3584 (36.31)	43 (30.50)	3627 (36.23)	
Other	7 (0.07)	0 (0.00)	7 (0.07)	
**Sexual orientation**				<.0001
Heterosexual	960 (9.73)	43 (30.50)	1003 (10.02)	
Lesbian/Gay/Bisexual	72 (0.73)	5 (3.55)	77 (0.77)	
Other	5 (0.05)	0 (0.00)	5 (0.05)	
Missing	8833 (89.49)	93 (65.96)	8926 (89.16)	
**Primary language**				1.00
English	9769 (98.98)	140 (99.29)	9909 (98.98)	
Spanish	93 (0.94)	1 (0.71)	94 (0.94)	
Other	8 (0.08)	0 (0.00)	8 (0.08)	
**Involvement in the criminal justice system**				0.520
No	4609 (46.70)	62 (43.97)	4671 (46.66)	
Yes	5261 (53.30)	79 (56.03)	5340 (53.34)	
**Medi-Cal beneficiary**				0.003
No^c^	1255 (12.72)	6 (4.26)	1261 (12.60)	
Yes	8613 (87.26)	135 (95.74)	8748 (87.38)	
Missing	2 (0.02)	0 (0.00)	2 (0.02)	
**Primary substance used**				0.193
Alcohol	2788 (28.25)	41 (29.08)	2829 (28.26)	
Stimulant	3940 (39.92)	54 (38.30)	3994 (39.90)	
Opioid	2400 (24.32)	34 (24.11)	2434 (24.31)	
Marijuana	576 (5.84)	6 (4.26)	582 (5.81)	
Other	166 (1.68)	6 (4.26)	172 (1.72)	
**Secondary substance used**				0.006
Alcohol	1167 (11.82)	17 (12.06)	1184 (11.83)	
Stimulant	2439 (24.71)	35 (24.82)	2474 (24.71)	
Opioid	1007 (10.20)	28 (19.86)	1035 (10.34)	
Marijuana	1783 (18.06)	25 (17.73)	1808 (18.06)	
Other	3473 (35.19)	36 (25.53)	3509 (35.05)	
Missing	1 (0.01)	0 (0.00)	1 (0.01)	
**Has a co-occurring mental health diagnosis**				0.556
No	4146 (42.01)	60 (42.55)	4206 (42.01)	
Yes	5686 (57.61)	80 (56.74)	5766 (57.60)	
Missing	38 (0.39)	1 (0.71)	39 (0.39)	
**Source of referral**				0.004
Criminal justice system involved	2937 (29.76)	40 (28.37)	2977 (29.74)	
Child protective services	133 (1.35)	3 (2.13)	136 (1.36)	
Drug treatment program	1135 (11.50)	21 (14.89)	1156 (11.55)	
Personal referral	5110 (51.77)	59 (41.84)	5169 (51.63)	
Other community resource	555 (5.62)	18 (12.77)	573 (5.72)	
**Organization-level characteristics**				
**Current level of care**				<.0001
Residential	7203 (72.98)	59 (41.84)	7262 (72.54)	
Withdrawal Management	2667 (27.02)	82 (58.16)	2749 (27.46)	
**Type of treatment service**				<.0001
Residential detoxification (non-hospital)	2664 (26.99)	81 (57.45)	2745 (27.42)	
Residential treatment/recovery	7206 (73.01)	60 (42.55)	7266 (72.58)	
**Outcomes**				
**Connection status**				0.005
Connected to care	7070 (71.63)	116 (82.27)	7186 (71.78)	
Not connected to care	2800 (28.37)	25 (17.73)	2825 (28.22)	
**Timely connection**				0.033
≤10 days	3554 (36.01)	65 (46.10)	3619 (36.15)	
> 10 days	3233 (32.76)	35 (24.82)	3268 (32.64)	
Missing	3083 (31.24)	41 (29.08)	3124 (31.21)	

a Number of observations and % of observations.

b P-value estimated from Students *t*-test for continuous variables and chi-square and Fisher’s exact tests (for variables with cells with <5 observations) for categorical variables.

c Those who were not Medi-Cal beneficiaries largely uninsured.

### 1.2 Study aim

This quasi-experimental study examines the pragmatic effectiveness of the MEET intervention for improving the likelihood of successful and timely connection to a lower level of care for individuals discharged from a SUD treatment program.

## 2 methods

### 2.1 Study context

This study was conducted within the San Diego County Behavioral Health Services (SDCBHS) Drug Medi-Cal Organized Delivery System (DMC-ODS). SDCBHS provides mental health and SUD treatment services within the fifth most populous county in the U.S. (U.S. Census Bureau, 2023). The DMC-ODS initiative provides a continuum of medically necessary SUD treatment services to Medi-Cal beneficiaries, modeled after the American Society of Addiction Medicine (ASAM) Criteria for SUD treatment services (Gastfriend and Mee-Lee, 2004). The ASAM Criteria are established standards to assist practitioners in determining the levels of care for individual SUD treatment needs. The ASAM levels of care in the SDCBHS DMC-ODS at the time of this study were defined as Level 1 – Outpatient, Level 2 – Intensive Outpatient, Level 3 – Residential, and Level 4 – Intensive Inpatient/Withdrawal Management. Individuals participating in this study were receiving services at Levels 3 or 4, with the goal of effectively stepping down from Level 4 to Level 3 or from Level 3 to Levels 2 or 1.

In 2020, SDCBHS initiated a SUD performance improvement project (PIP) aiming to improve connections to care as individuals stepped down from inpatient and residential treatment programs to outpatient levels of care. The PIP evaluation team selected the MEET intervention for this quality improvement initiative given its potential impacts on the SDCBHS SUD system and its prior successful application to increase treatment engagement in the child, youth, and family portions of SDCBHS.

### 2.2 Design and implementation of the intervention

This study assessed the effectiveness of the MEET intervention to improve care continuity and timely care connections for clients discharged from inpatient and residential DMC-ODS SUD treatment programs with referrals to a lower level of care. Leaders of programs within the SDCBHS DMC-ODS with the lowest timely connection rates were initially identified by the county and invited to participate in training and implementation of the MEET intervention. These targeted invitations were made in an attempted to engage programs with lower connection rates for overall system improvement. Targeted invitations were followed by an open invitation to other leaders of inpatient and residential SUD treatment programs. A total of five programs (7 % of total SUD programs, 18 % of total withdrawal management and residential SUD programs) participated in the PIP and are included in the present study, four of which were selected based on existing low connection rates, and the fifth program identified via open invitation.

Program leaders and providers participated in a virtual group training led by the first author, which included guidance on engagement, MI, anticipating barriers and the use of MEET in the context of treatment initiation, planning, and preparation for discharge. Service program teams then developed individualized implementation plans with goals, objectives, and action steps. With support from the first, third, and fourth authors, training attendees identified key individuals involved in the action steps, anticipated resources and timeframes, considered potential barriers and indicators of measurable progress.

The implementation approach for MEET was informed by the Exploration, Preparation, Implementation, and Sustainment (EPIS) framework (Aarons et al., 2011; Moullin et al., 2019), a widely used implementation science framework applied to diverse public care contexts. EPIS delineates four linked phases of implementation specified in the framework’s name and key domains (e.g., outer context, inner context) that influence implementation. Implementation planning was included as part of the preparation phase, encouraging use of implementation strategies to support the integration and delivery of MEET.

Each program also identified an implementation champion and an individual responsible for oversight of the implementation plan and engaged in group training and ongoing post-training consultation for nine months during the implementation phase (February 2021 through November 2021). This workgroup, including the implementation champions, Contracting Officer Representatives, SDCBHS evaluators, a research team from UC San Diego and the first author, met bi-monthly to help guide the study and provide a platform for champions to discuss their progress, successes and barriers. The group provided ongoing feedback during the consultations and data collection was used to inform adaptations and support successful implementation. Often, the workgroup assisted with aligning SDCBHS administrative structures and new workflows, as practitioners utilized the intervention collaboratively with clients in their treatment planning, discharge preparation, and the transition of care between programs. For example, programs received ongoing support to align the MEET intervention with billing mechanisms and best practices for clients transitions during the consultation period.

Primarily Certified Alcohol and Drug Counselors, Certified SUD Counselors, and Registered Alcohol and Drug Technicians delivered the MEET intervention, although in some cases other behavioral health providers (i.e., behavioral health therapists) delivered the intervention. The intervention was intended to be applied once per client in a single session with the provider at the time of transition planning; however, some programs divided MEET into more than one session to align to their discharge planning practices. The timing of the transition planning visit and MEET intervention during the client’s treatment plan was dependent on the client’s length of stay and readiness. Information from intervention sessions was documented in a transition plan, which remained in the client’s medical record to encourage consistency across providers within and across programs before and after discharge.

Clients included in this study were clients admitted to a Residential or Intensive Inpatient/Withdrawal Management SUD treatment program and were candidates for discharge because they (a) completed treatment, (b) met their recovery goals and (c) were referred to continued treatment. Clients meeting these criteria between July 2020 and January 2022 were included in the study. The UC San Diego Institutional Review Board approved this study.

### 2.3 Data collection

Intervention data was documented by the intervention providers during implementation of MEET in the form of a discharge planning document. Program leaders provided copies of these documents to the PIP evaluation team at regular intervals to monitor implementation and utilization of the intervention over the course of the PIP. These data were merged with SUD treatment and client demographic data collected from the SDCBHS DMC-ODS electronic health record (EHR) system.

### 2.4 Measures

Our primary exposure of interest was receipt of the MEET intervention, which was a dichotomous variable indicating whether a client received the intervention or not.

Our primary outcome of interest was connection to SUD treatment, defined as a client’s admission to a lower ASAM level of care following discharge from a treatment program. This was assessed by the presence of a discharge and subsequent admission date for each level of care in the EHR. Our secondary outcome measured whether the admission date to the lower level of care occurred within (≤) 10 calendar days of the discharge from the referring program. We defined timely connection as a documented admission date within 10 calendar days of a documented discharge date for consistency with standardized evaluation reporting conducted by SDCBHS.

Covariates of interest included characteristics at the individual- and organizational-levels and were obtained from the EHR. Selection of covariates was based on their established relevance in the literature and their potential impact on the primary and secondary outcomes of interest. Individual-level characteristics included sociodemographics (age, race/ethnicity, gender, sexual orientation, primary language spoken, involvement in the criminal justice system, Medi-Cal beneficiary status), and clinical factors (primary and secondary substance used and co-occurring mental health diagnosis). Organization-level characteristics included the client’s level of care at the time of the evaluation (residential or withdrawal management), the type of treatment service received (detoxification or residential treatment/recovery), and their source of referral to the SDCBHS SUD treatment program.

### 2.5 Statistical analysis

We used directed acyclic graphs (DAGs) to depict known or plausible causal relationships between our intervention, outcomes of interest, and hypothesized relevant covariates, which informed the selection of variables in our analysis (see Supplemental Figure 1; Greenland, Pearl, Robins, 1999). We calculated descriptive statistics to characterize our sample by intervention status. Continuous variables are presented as mean and standard deviation (SD), and categorical variables are reported as frequencies and percentages. Intervention groups were compared using Student’s *t*-test and Chi-square or Fisher’s exact tests for continuous and categorical variables as appropriate, respectively.

Adjusted odds ratios (AOR) were estimated by logistic regression models for the probability of each outcome with an indicator for intervention status (i.e., MEET or no MEET). Models were estimated via generalized estimating equations (GEE) and an exchangeable covariance matrix to account for clustering within treatment facilities and individuals. Stabilized inverse probability of treatment weighting (IPTW) was used to adjust for baseline confounding. Informally, the denominator of these weights is the probability that an individual received their observed intervention given their baseline covariates. Use of these weights creates a pseudo-population in which receipt of MEET is independent of the measured confounders (Austin and Stuart, 2015). The denominator was estimated using a logistic regression model for the probability of receiving MEET given covariates, which included age, race/ethnicity, gender, primary language, involvement in the criminal justice system, Medi-Cal beneficiary status, primary and secondary substance used, co-occurring mental health diagnosis, and source of referral. Covariates for the IPTW model were selected *a priori* based on the literature and refined through team meetings using DAGs to represent plausible causal relationships between the intervention, outcomes, and relevant covariates (Curran et al., 2009, Lind et al., 2019, Lappan et al., 2020). However, we decided not to include sexual orientation, which was included our DAG, in our IPTW calculation due to significant (~90 %), likely structural, missingness on that variable. We considered the same covariates to be confounders of both our primary and secondary outcomes and thus applied the same IPTW to each outcome model. Descriptive statistics for variables included in our IPTW model post-IPTW application are included in Supplemental Table 1. Interpretations are based on the magnitude of the AORs and corresponding 95 % confidence intervals and were used to report the effect of MEET on primary and secondary outcomes, respectively (Sterne and Smith, 2001, Amrhein et al., 2019). All DAGs were developed using DAGitty.net and statistical analyses were conducted using SAS version 9.4 (SAS Institute, Inc.; Cary, NC).

Due to substantial missingness (>30 %) in our secondary outcome (days to connection), we performed a sensitivity analysis in which any missing value for ‘days to connection’ was re-categorized as > 10 days. Re-categorizing missingness in this way ensured we were modeling the most conservative outcome. Results from this sensitivity analysis are reported in Supplemental Table 2.

## 3 Results

### 3.1 Sample characteristics

Participants had a mean age of 36.8 years (SD: 11.4), and most were White (50.5 %), cisgender men (63.7 %) who primarily spoke English (99.0 %). Most were previously involved in the criminal justice system (53.3 %) and Medi-Cal beneficiaries (87.4 %). Many participants primarily used stimulants (39.9 %), alcohol (28.3 %), and opioids (24.3 %), and secondarily used stimulants (24.7 %) or ‘Other’ drugs (35.1 %). A majority had a co-occurring mental health diagnosis (57.6 %) and were currently in residential treatment (72.5 %) and receiving residential treatment/recovery services (72.6 %). Over half were referred to their current treatment facility through a personal referral (51.6 %). While only 1.4 % received the MEET intervention, nearly 75 % of the total sample was connected to care and 36.2 % were connected to care in ≤ 10 days.

Participants who received MEET appeared more likely to be White (p = 0.026), heterosexual (p < 0.0001), Medi-Cal beneficiaries (p = 0.003), to use opioids as their secondary substance (p = 0.003) and be in withdrawal management (p < 0.0001) receiving residential detoxification (p < 0.0001). Also, participants who received MEET appeared more likely to be referred to treatment by an ‘other community resource’ (p = 0.004). Those who received MEET were more likely to be connected to care (p = 0.005) and have a ‘timely connection’ in ≤ 10 days (p = 0.033).

### 3.2 Primary and secondary outcomes

In separate IPTW weighted models, participants who received the MEET intervention had 1.79 times the odds of being connected to a lower-level of SUD treatment compared to those who did not receive MEET (AOR=1.79, 95 % confidence interval [CI]: 1.11, 2.90), and participants who received MEET also had 1.65 times the odds of being connected to a lower-level of SUD treatment within 10 days compared to those who did not receive MEET (AOR=1.65, 95 % CI: 1.01, 2.70).

## 4 Discussion

The current work reports findings from the evaluation of the MEET intervention on enhancing successful and timely service connection for individuals transitioning between SUD treatment settings within the context of a community-initiated PIP. As part of this PIP, five SUD treatment programs received training in and implemented the MEET intervention. Our findings indicate that MEET enhanced successful SUD treatment program transitions, with MEET recipients having nearly twice the odds of timely connection. Overall, our preliminary effects of the MEET intervention on improving timely service connection highlight its potential as an effective, real-world intervention for improving SUD service utilization and outcomes.

As Baker et al., (2020) point out in their study of long-term residential SUD treatment retention, while there are many unmodifiable risk factors for attrition, leveraging the modifiable predictors of perseverance in treatment can lead to a much better return on the enormous social and economic investments made in SUD treatment (Cartwright, 2000). The MEET intervention offers a structured strategy to leverage motivation and engagement and to elicit personalized treatment planning and discharge preparation from individuals participating in SUD treatment services. Our findings add to the expanding study of effective interventions targeting SUD care transitions (James et al., 2023, Incze et al., 2024, Krawczyk et al., 2023, Treitler et al., 2024), particularly with the use of MI-based strategies (Carroll et al., 2006).

Importantly, MEET was implemented and evaluated within community-based SUD treatment programs as part of a community-initiated PIP, with programs identified based on their need for enhancing care connections versus specific program characteristics. As a result, enrolled programs were diverse, with clients both receiving and referred for various services and care types (e.g., detoxification, residential treatment/recovery). Our results suggest that MEET effectively enhanced timely connection even after covarying for these organizational characteristics. Thus, our results speak to the practical effectiveness of MEET when implemented in real-world settings, where the nature and types of SUD treatments commonly vary.

Several aspects considered during the development and roll-out of MEET likely supported the successful uptake and demonstrated preliminary effectiveness we observed within community settings. MEET was designed for community implementation, including during its initial development and its subsequent adaptation for scale-up to additional service contexts, supporting its feasibility in community settings such as SUD treatment programs. Designing for community implementation and dissemination by considering the fit of an intervention with the context, system, practitioner, and recipient during development is critical for successful translation to community settings (Brownson et al., 2013, Kwan et al., 2022).

In addition to the design of MEET, which was informed by EPIS, there was significant and intentional preparation for the implementation of MEET within SUD treatment programs. Preparation consisted of community-partnered implementation plan development which specified strategies to support MEET implementation, including identification of implementation champions, training paired with ongoing coaching, ensuring the MEET intervention aligned with program and broader system procedures and funding requirements and resources to ensure its feasibility and sustainability. Intentional and proactive preparation for implementation that considers and addresses determinants within the inner organizational and outer system contexts is essential to enhancing implementation and associated effectiveness outcomes (Aarons et al., 2011, Moullin et al., 2019). While beyond the scope of the current work, further evaluation of the factors associated with the delivery or implementation of the MEET intervention as well as implementation outcomes such as fidelity and acceptability of MEET are planned.

### 4.1 Limitations

While the MEET intervention showed promising results, there are several limitations to consider in the context of this study. First, relatively few participants received MEET, which may have affected the robustness of analyses. The small sample size of the intervention group likely reduced our ability to detect meaningful differences, leading to null results in the presence of actual effects. However, our magnitudes of effect were large, lending credence to our findings. Second, our study had substantial missingness on our secondary outcome (days to connection) and sexual orientation, the latter of which is a potentially important confounder of the relationship between MEET and a successful SUD care transition. Although we performed a sensitivity analysis on our secondary outcome to categorize missing values conservatively, the missing data might still have introduced bias or reduced the precision of results. Additionally, while IPTW helped to control for confounding, it relies on the assumption that all relevant confounders were measured and accounted for. If unmeasured confounding exists, such as with sexual orientation, the results could still be biased. For example, we were unable to control for provider-level variations in intervention delivery (i.e., fidelity) or participant motivations, which could have influenced results. Future randomized controlled trials may overcome this limitation and likely are a meaningful next step in the evaluation of MEET’s efficacy. Third, due to the real-world conditions of this study, participating programs were not selected randomly, but instead through targeted invitations based on low connection rates and then through open invitations. Four of the five programs were identified as having low connection rates, which raises the possibility for bias based on the selection procedures. We controlled for clustering at the program and participant level, therefore we can assume program-level variance would not explain our results. However, since programs with the lowest connection rates were most strongly represented in the findings, MEET may be most effective for programs with low connection rates or where overall performance is farther from the desired goal. Future randomized studies would be needed to evaluate further. Finally, this work was conducted within one county-funded SUD system in only a handful of treatment programs, with inherently limited generalizability for settings beyond the Southern California community setting where MEET was implemented or beyond Medi-Cal beneficiaries with previous criminal justice involvement who mainly comprise the participants in this study. Studying the intervention in broader and more diverse contexts and among other populations would strengthen confidence in these initial findings and would better elucidate the implementation determinants that may contribute to the effectiveness of MEET so it may be disseminated across different SUD treatment settings and levels of care. Future research should investigate how to optimize MEET's application across diverse contexts.

## 5 Conclusion

This study explored the impact of the MEET intervention on improving the continuity of care for individuals transitioning from higher to lower levels of SUD treatment within San Diego County's Drug Medi-Cal Organized Delivery System. Our results demonstrate that the MEET intervention significantly enhanced the likelihood of individuals being connected to follow-up care after discharge from intensive SUD treatment. Specifically, individuals who received the MEET intervention had 79 % increased odds of being connected to subsequent SUD treatment services compared to those who did not receive MEET. Moreover, these individuals were 65 % more likely to achieve a timely connection, defined as engaging in follow-up care within 10 days of discharge. These findings underscore the potential of MEET to address barriers to treatment retention by eliciting individual motivation and facilitating smoother transitions between care levels.

## CRediT authorship contribution statement

**Crandal Brent R.:** Writing – review & editing, Writing – original draft, Methodology, Investigation, Conceptualization. **Eger William H.:** Writing – review & editing, Writing – original draft, Methodology, Formal analysis. **Panczakiewicz Amy:** Writing – review & editing, Writing – original draft, Validation, Project administration, Investigation, Conceptualization. **Hillery Naomi:** Writing – review & editing, Validation, Project administration, Investigation, Data curation, Conceptualization. **Arriola Freddy:** Validation, Investigation, Data curation. **Xu Zhun:** Validation, Investigation, Data curation. **Dickson Kelsey S.:** Writing – review & editing, Writing – original draft, Methodology, Investigation, Formal analysis, Conceptualization.

## Declaration of Competing Interest

The authors declare that they have no known competing financial interests or personal relationships that could have appeared to influence the work reported in this paper

## Footnotes

Appendix A Supplementary data associated with this article can be found in the online version at doi:10.1016/j.dadr.2025.100332.
