# Supplementary material for: Real-world effectiveness of Motivational Enhancement for Engagement in Treatment (MEET) to improve substance use disorder care transitions
Source: Drug Alcohol Depend Rep. 2025 Apr 11;15:100332. doi: 10.1016/j.dadr.2025.100332 (PMC12433798; doi:10.1016/j.dadr.2025.100332)
Supplement: Supplementary file 1 — Supplementary material [file mmc1.docx]

**Supplemental Figure 1. Directed Acyclic Graph (DAG) used to determine characteristics included in inverse probability of treatment weighting.**

*
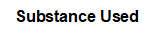
***
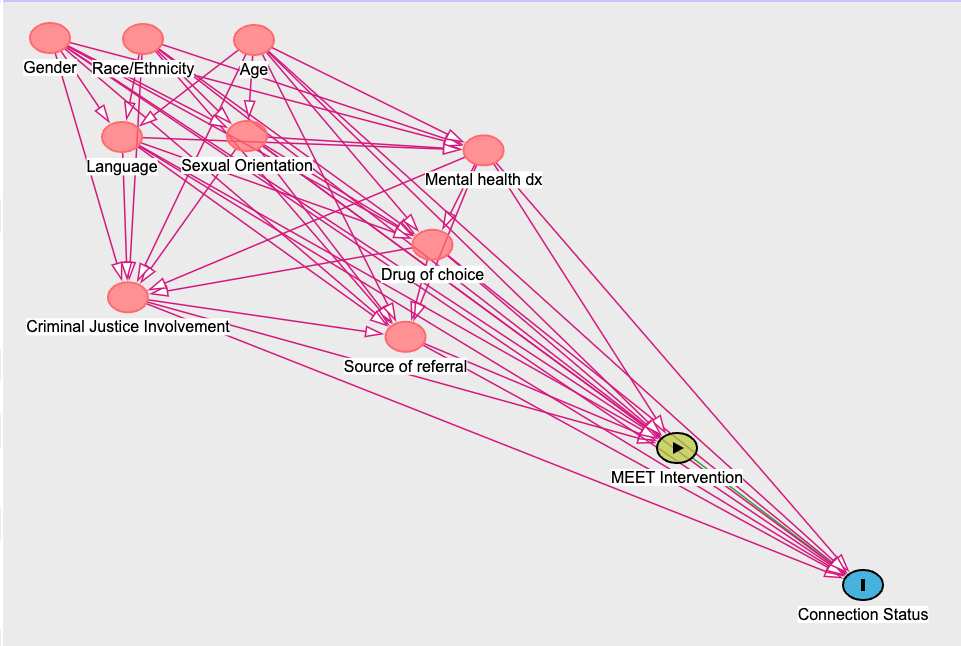
**

*Notes.* This DAG shows hypothesized relationships among our intervention (MEET), our primary outcome (Connection Status) and confounders of interest (age, race/ethnicity, gender, primary language, involvement in the criminal justice system, Medi-Cal beneficiary status, primary and secondary substance used, co-occurring mental health diagnosis, and source of referral).

**Supplemental Table 1. Baseline characteristics weighted by individual’s inverse probability of treatment weight (IPTW).**

|  | **Did not receive MEET**  **(n=9,870.77; 98.60)^a^** | **Received MEET**  **(n=140.43; 1.40)** | **Overall**  **(N=10,010.2; 100.00)** |
| --- | --- | --- | --- |
| **Individual-level characteristics** |  |  |  |
| **Mean age in years (SD)** | 36.79 (11.40) | 37.54 (10.99) | 36.80 (11.39) |
| **Race/Ethnicity** |  |  |  |
| White | 4979.76 (50.45) | 70.84 (50.45) | 5050.60 (50.45) |
| Hispanic | 2976.37 (30.16) | 42.74 (30.44) | 3019.11 (30.16) |
| Black/African American | 1034.22 (10.48) | 15.83 (11.27) | 1050.05 (10.49) |
| Other | 875.48 (8.87) | 11.02 (7.85) | 886.49 (8.86) |
| Missing | 3.94 (0.04) | 0 (0.00) | 3.94 (0.04) |
| **Gender** |  |  |  |
| Cisgender man | 6287.06 (63.70) | 88.15 (62.77) | 6375.21 (63.69) |
| Cisgender woman | 3575.81 (36.23) | 52.28 (37.23) | 3628.09 (36.24) |
| Other | 6.90 (0.07) | 0 (0.00) | 6.90 (0.07) |
| **Primary language** |  |  |  |
| English | 9769.24 (98.98) | 140.13 (99.79) | 9909.38 (98.99) |
| Spanish | 92.64 (0.94) | 0.29 (0.21) | 92.93 (0.93) |
| Other | 7.89 (0.08) | 0 (0.00) | 7.89 (0.08) |
| **Involvement in the criminal justice system** |  |  |  |
| No | 4604.97 (46.66) | 66.00 (47.00) | 4670.97 (46.66) |
| Yes | 5264.80 (53.34) | 74.43 (53.00) | 5339.23 (53.34) |
| **Medi-Cal beneficiary** |  |  |  |
| No | 1243.22 (12.60) | 16.59 (11.82) | 1259.82 (12.59) |
| Yes | 8624.57 (87.38) | 123.83 (88.18) | 8748.41 (87.39) |
| Missing | 1.97 (0.02) | 0 (0.00) | 1.97 (0.02) |
| **Primary substance used** |  |  |  |
| Alcohol | 2789.16 (28.26) | 43.99 (31.33) | 2833.15 (28.30) |
| Stimulant | 3937.73 (39.90) | 55.95 (39.84) | 3993.68 (39.90) |
| Opioid | 2399.70 (24.31) | 32.59 (23.21) | 2432.28 (24.30) |
| Marijuana | 573.80 (5.81) | 6.71 (4.78) | 580.51 (5.80) |
| Other | 169.39 (1.72) | 1.18 (0.84) | 170.58 (1.70) |
| **Secondary substance used** |  |  |  |
| Alcohol | 1167.25 (11.83) | 18.61 (13.25) | 1185.86 (11.85) |
| Stimulant | 2439.08 (24.71) | 31.77 (22.62) | 2470.84 (24.68) |
| Opioid | 1020.38 (10.34) | 16.21 (11.54) | 1036.59 (10.36) |
| Marijuana | 1782.58 (18.06) | 26.83 (19.11) | 1809.41 (18.08) |
| Other | 3459.50 (35.05) | 47.01 (33.47) | 3506.50 (35.03) |
| Missing | 0.99 (0.01) | 0 (0.00) | 0.99 (0.01) |
| **Has a co-occurring mental health diagnosis** |  |  |  |
| No | 4146.67 (42.01) | 54.22 (38.61) | 4200.89 (41.97) |
| Yes | 5684.66 (57.60) | 85.91 (61.18) | 5770.58 (57.65) |
| Missing | 38.44 (0.39) | 0.29 (0.21) | 38.73 (0.39) |
| **Source of referral** |  |  |  |
| Criminal justice system involved | 2935.07 (29.74) | 41.15 (29.31) | 2976.22 (29.73) |
| Child protective services | 134.08 (1.36) | 1.54 (1.10) | 135.62 (1.35) |
| Drug treatment program | 1139.76 (11.55) | 17.00 (12.11) | 1156.76 (11.56) |
| Personal referral | 5096.08 (51.63) | 71.77 (51.11) | 5167.86 (51.63) |
| Other community resource | 564.78 (5.72) | 8.96 (6.38) | 573.75 (5.73) |

^a^Number of observations and % of observations.

**Supplemental Table 2. Association between receiving MEET and Days to Connection.^$^**

|  | **>10 days**  **(n=6392)**  **AOR (95% CI)^a^** | ≤**10 days**  **(n=3619)**  **AOR (95% CI)^a^** |
| --- | --- | --- |
|  |  |  |
| Received MEET^b^ | REF | 1.49 (1.00, 2.21) |

^a^ AOR (95% CI) = Adjusted odds ratio and 95% confidence interval for the marginal effect of receiving the intervention (MEET) on the likelihood of connection to addiction treatment, estimated using a weighted generalized estimating equations (GEE) model, adjusting for clustering within treatment facilities and individuals.

^b^ Model weight accounts for age, race/ethnicity, gender, primary language, involvement in the criminal justice system, Medi-Cal beneficiary status, primary and secondary substance used, co-occurring mental health diagnosis, and source of referral.

^$^ Where any missing value for days to connection is modeled as >10 days (i.e., the most conservative scenario).
